# First Glimpse at the Diverse Aquaporins of Amphipod Crustaceans

**DOI:** 10.3390/cells10123417

**Published:** 2021-12-04

**Authors:** Andrea Desiderato, Tomasz Mamos, Tomasz Rewicz, Artur Burzynski, Serena Mucciolo

**Affiliations:** 1 Department of Invertebrate Zoology and Hydrobiology, University of Lodz, Banacha 12/16, 90-237 Łódź, Poland; tomasz.mamos@biol.uni.lodz.pl (T.M.); tomasz.rewicz@biol.uni.lodz.pl (T.R.); serena.mucciolo@biol.uni.lodz.pl (S.M.); 2 Department of Genetics and Marine Biotechnology, Polish Academy of Sciences, Institute of Oceanology, Powstańców Warszawy 55, 81-712 Sopot, Poland; aburzynski@iopan.pl

**Keywords:** Aqp8, Prip, Bib, Aqp12, Glp, amphipods, in silico, evolution

## Abstract

The importance of aquaporins (AQPs) in the transport of water and solutes through cell membranes is well recognized despite being relatively new. To date, despite their abundance, diversity, and presence in disparate environments, amphipods have only been mentioned in studies about the AQPs of other animals and have never been further investigated. In this work, we aimed to recover from public data available AQPs of these crustaceans and reconstruct phylogenetic affinities. We first performed BLAST searches with several queries of diverse taxa against different NCBI databases. Then, we selected the clades of AQPs retrieving the amphipod superfamily Gammaroidea as monophyletic and ran phylogenetic analyses to assess their performances. Our results show how most of the AQPs of amphipods are similar to those of other crustaceans, despite the Prip-like displayed different paralogs, and report for the first time a putative Aqp8-like for arthropods. We also found that the candidate genes of Prip-like, Bib-like, Aqp12-like, and Glp-like help solve deeper relationships in phylogenies of amphipods while leaving uncertainties in shallower parts. With our findings, we hope to increase attention to the study of amphipods as models for AQP functioning and evolution.

## 1. Introduction

Aquaporins (AQPs) are transmembrane proteins present in almost all living organisms that facilitate the passive transport of mainly water and/or other small solutes, such as glycerol, ammonia, metalloids, and carbon dioxide [1,2]. They are assembled in homotetramers, forming a five-pore quaternary structure, and each monomer is composed of six transmembrane domains containing a distinct pore [3]. The two main restrictions are the Asn-Pro-Ala (NPA) motifs, which are involved in proton and cation exclusion, and the ar/R selectivity filter (three aromatic amino acids and one arginine), which represents the narrowest part of the protein and affects substrate selectivity [4,5,6,7]. Despite these shared structural features, their overall primary structure is poorly conserved, sharing only ~30% identity [8]. Reflecting the phylogenetic reconstructions of the different AQPs, these proteins have been divided into four main groups (all belonging to the membrane intrinsic protein group—MIPs): (i) Aqp1-like, the classical or orthodox AQPs permeable mainly to water and/or small molecules; (ii) Aqp8-like, the aquaammoniaporins permeable to ammonia and other small molecules; (iii) Aqp3-like (hereafter referred to as Glp-like) the aquaglyceroporins involved mostly in the transit of the glycerol and/or water and other small molecules; (iv) Aqp11-like, the superaquaporins, also called unorthodox AQPs both intracellular, largely diverging in sequence and function from the previous AQPs [9,10].

Among the invertebrates, arthropods are one of the few taxa in which AQPs were studied. However, most of the works are focused on hematophagous ectoparasites, such as mosquitos and ticks, acting as vectors of bacterial and viral pathogens (e.g., [11,12,13,14]). Recently, the AQPs of the salmon louse *Leopeophtheirus salmonis salmonis* (Krøyer, 1837) (hereafter referred to as *L. salmonis*) and the barnacle *Amphibalanus improvisus* (Darwin, 1854) were recovered, functionally characterized, and compared with the publicly available sequences of other arthropods [15,16]. A total of six main subfamilies were identified in this phylum: Drip, Prip, Bib, Eglp—all exclusive of arthropods and belonging to the Aqp1-like group, Glps (Aqp3-like) and Aqp12L (Aqp11-like) [17]. No aquaammoniaporins were recovered [15,16]. Drip and Prip proteins are mainly involved in water transit [18,19], Bib is permeable to uncharged gases [20], and Eglp—only occurring in insects, such as Drip, which is permeable mostly to glycerol [21]. In crustaceans, the involvement of AQPs in osmoregulation was pointed out by their transcriptional regulation in response to salinity stress (e.g., [15,22,23]). Moreover, aquaglyceroporins may have a relevant anti-freezing role, controlling the glycerol contents in various tissues during winter [15].

In the diverse crustacean class Malacostraca, the order Amphipoda includes species inhabiting various environments, including freshwater, brackish, marine, and terrestrial ones. Their known diversity currently exceeds 10,400 species [24], of which approximately 20% are freshwater species [25]. They are often abundant in local ecosystems and extremely important for the circulation of organic matter (i.e., [26]), as well as being an essential component in the diet of fishes, birds, and other organisms [27]. Some amphipods are widely distributed and used as model species in numerous evolutionary studies (e.g., [28,29]), frequently showing high levels of cryptic diversity [30,31] even when occurring sympatrically [32,33]. Being generally sensitive to pollutants such as heavy metals or oil, they are often used as bioindicators in ecotoxicological studies [34,35,36], although cryptic lineages might differ in their tolerance to contaminants [37], necessitating molecular approaches (e.g., DNA barcoding) to avoid biased results [38]. Nevertheless, many species are recognized as successful invaders worldwide [39,40], disrupting ecosystem functioning in both marine [41] and freshwater environments [42]. To date, no study concerning amphipod AQPs has been carried out, and the only reports on the presence of some gene subfamilies have been indirectly addressed in works related to *L. salmonis* [16,43] but never further explored.

In this work, we aimed to investigate, through an *in silico* approach, the presence and diversity of possible candidates of different AQP groups in amphipod crustaceans then, using the diverse superfamily Gammaroidea as a case study to explore the possible application of AQPs in phylogenetic studies.

## 2. Materials and Methods

To verify the presence of every putative AQP main group, protein sequences of each human AQP (i.e., Aqp1, Aqp8, Aqp11, Aqp3; Supplementary Table S1) were used as queries to perform BLASTP and TBLASTN searches on various online databases available in NCBI (nr, proteins, genomes, assembly, SRA, TSA, WGS) between July and September 2021. Additionally, the AQP nucleotide sequences of *Leopeophtheirus salmonis* were used as a query in the same databases using BLASTX and TBLASTX. Afterward, the best matches of annotated amphipods (i.e., *Trinorchestia longiramus* Jo, 1988 and *Hyalella azteca* (Saussure, 1858)) were included as queries to maximize the recovery of putative AQPs. Only BLAST hits with expectation values (e-values) lower than 1e-15 and query coverage of 40% were considered. The corresponding nucleotide sequences were trimmed, and when necessary, the fragments were concatenated to construct a coding sequence (i.e., SRA, WGS). Then, all the sequences resulting from the same AQP-BLAST (e.g., Glp-like, Aqp8-like) were translated, aligned using MAFFT v7.45 [44] in GENEIOUS 11 [45], manually checked and modified. The single alignments were then merged, and possible duplicates resulting from different BLASTs (e.g., Prip-like from *L. salmonis* and Aqp1 of humans) were removed. Additionally, the dataset was integrated using *de novo* assemblies of transcriptomes done in [46]. The obtained transcriptomes were functionally annotated following methodology from [47]. The AQPs in this dataset were found in assemblies for *Baikalogammarus pullus* (Dybowsky, 1874), *Echinogammarus berilloni* (Catta, 1878), *Marinogammarus marinus* (Leach, 1815), *Gammarus wautieri* A. L. Roux, 1967, *G. pulex* (Linnaeus, 1758), and *Obesogammarus crassus* (Dybowsky, 1874). Only non-redundant sequences were selected and used for phylogenetic analyses, and information about identical sequences can be accessed in Supplementary Table S1. The alignment was complemented with AQP sequences of humans, *L. salmonis, A. improvisus* (these species were chosen because of the recent studies describing their AQPs), decapods, and insects, to have a proper representation of the already known AQPs and the relative position of the amphipod ones. For the same reason, some sequences of the bacterial AqpZ, as well as Eglp and Drip, were included. Information about all the sequences used can be found in Supplementary Table S1, as well as the complete alignment of nonredundant translated sequences (Alignment S1).

With this alignment (446 sequences and 469 positions), a maximum likelihood (ML) was inferred with PhyML [48] using an approximate likelihood ratio test (aLRT) to estimate the support of the branches [49]. The best substitution model (LG + I + G_4_) was selected with the SMS routine in PhyML using the Bayesian information criterion (BIC) [50]. In the ML phylogeny of the deduced amino acids, we identified the clades of AQPs where the superfamily Gammaroidea (Amphipoda) was recovered as monophyletic. Then, one nucleotide sequence/haplotype per species for each AQP main group (i.e., Prip-like1, Bib-like, Aqp12-like, and Glp-like) was selected, and an alignment for each putative protein was manually refined. To calibrate the phylogeny, sequences of cytochrome c oxidase subunit 1 (COX1) from NCBI were retrieved using the same procedure described above and using the complete gene of *B. pullus* as a query [46]. All the sequence information and the alignments can be found in Supplementary Table S2 and Alignments S2–S6.

For visualization of relationships within each gene and assessment of potential discrepancies in phylogenetic signals between genes, phylogenetic reconstructions of nucleotide alignments were performed employing the ML approach through RAxML 8.2.12 [51]. The best-scoring ML tree was produced using the substitution model selected through the SMS routine. Statistical support was estimated with thorough bootstrapping and automatic iteration determinant autoMR.

To estimate and visualize the temporal framework of the AQP genes of the superfamily Gammaroidea, a time-calibrated tree was constructed on the four selected AQP genes (i.e., Prip-like 1, Bib-like, Aqp12-like, and Glp-like) and the COX1 mtDNA gene, all treated as separate partitions, in BEAST 2.6.4 [52]. The molecular clock was calibrated using a substitution rate of 0.01773 [53], congruent with widely used rates, especially for gammarids [30,31,46,54,55]. As priors, the Birth-Death tree model and substitution model selected based on SMS determination were used. To obtain substitution rates relative to the COXI gene, all clock priors were set to strict. Four runs of the MCMC were performed, each 300 M generations long, and sampled every 30,000 generations. The results were examined for convergence in TRACER 1.7 [56]. All parameters in each run reached an effective sample size (ESS) above 100 and were combined using LogCombiner 2.6.4, removing 25% of iterations as the burn-in phase to provide an ESS above 200. The final tree was combined in LogCombiner 2.6.4 and summarized with Tree-Annotator 2.6.4. The combined logs were used to provide substitution rates for the four analyzed genes relative to COX1.

A smaller alignment of the amino acids with one amphipod sequence per clade of putative AQPs, including those of humans, *L. salmonis*, *A. improvisus*, *Pontastacus leptodactylus* (Eschscholtz, 1823), and *Carcinus maenas* (Linnaeus, 1758) (Supplementary Alignment S7), was used to create logos of the regions, including the ar/R and NPA motifs, using WebLogo (https://weblogo.berkeley.edu/, accessed on 10 October 2021). The tertiary structure of each putative AQP was predicted using Phyre2 [57] which implements homology detection methods to build 3D models, considering only the best hit models with a confidence of 100% and a coverage of at least 80% (except for the Bib, for which the max coverage recovered was 44%). Then, cartoon renderings of the proteins were produced in EzMol [58]. Finally, the prediction of structural changes introduced by single amino acid mutations was computed in Missense 3D [59].

## 3. Results and Discussion

We were able to retrieve and align a total of 611 amphipod sequences (only seven previously annotated as AQP), of which 401 nonredundant sequences belonged to 79 species of 18 different families and were grouped into five superfamilies (Supplementary Table S1). Gammaroid amphipods were the most abundant (71 species), followed by talitroids (six species), showing an important bias/gap in the genomic/transcriptomic data regarding these animals. Regardless, the finding of putative AQP sequences in these two superfamilies that inhabit marine, brackish, freshwater, and terrestrial environments, together with the deep-sea species *Hirondellea gigas* (Birstein and Vinogradov, 1955), shows that the expression of these proteins in relation to diverse environmental conditions might be a field deserving to be better explored.

### 3.1. Phylogeny of the Translated Sequences

**Figure 1:**
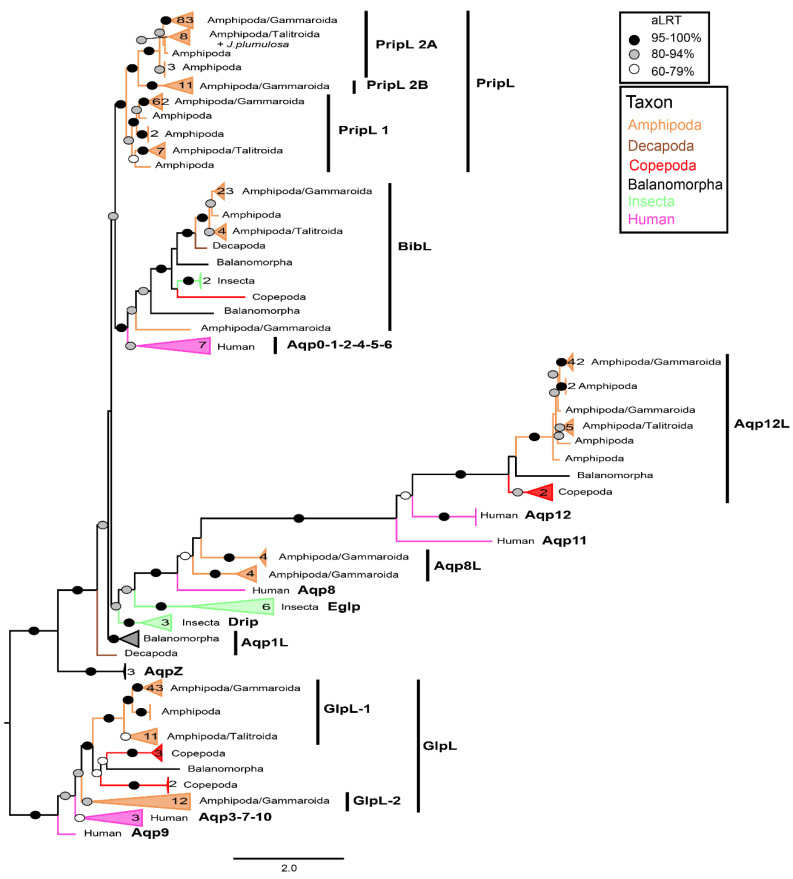
Maximum likelihood phylogeny of translated nonredundant sequences of AQPs. The number of sequences per collapsed clade reported beside it. Only aLRT support higher than 60% is shown (black: >95%; gray: 80–94%; white: 60–79%). Colors relative to the different taxa. The information relative to the sequences is available in Supplementary Table S1, as well as the complete alignment (Supplementary Alignment S1). See Supplement Figure S1 for the fully annotated phylogeny.

The general topology of the ML phylogeny of combined genes presented some discrepancies compared to other studies (e.g., [9]) such as the Aqp8-like clade paraphyletic and comprising the Aqp12-like one. This can be attributed to the long-branch attraction of the divergent Aqp12-like clade, but also to our choice of including a few sequences of other taxa. However, analyzing the relationships between different families and subfamilies of AQPs is out of the scope of this study as it is focused on the identification of different AQP candidates. Besides, it showed generally high support (>95%) for the clade of each AQP group (Figure 1). Interestingly, we retrieved sequences of putative amphipod Aqp8-like and Bib-like that were never recorded before. No Drip or Eglp were found, supporting hypothesis that these AQPs are found only in insects [16]. Gammaroids displayed three clades of possible Prip-like genes (i.e., Prip-like 1-2A-2B, with Prip-like 2A including also other amphipods), two putative Aqp8-like genes, and two Glp-like genes (i.e., Glp-like 1-2), showing the presence of many paralogs in each clade (Aqp12-like excluded). Moreover, one singleton of *Gammarus fossarum*, clustered in the general clade of Bib proteins (i.e., sequence 211, accession: GHCY01378359; see Supplementary Table S1), appeared more similar to Bib-like 2 of *Amphibalanus improvisus* (i.e., different from the normal Bib [15]). Nevertheless, this sequence was not included in successive analyses, as it might be a pseudogene.

### 3.2. In Silico 3D Structures and Conserved Regions in Gammaroid AQPs

**Figure 2:**
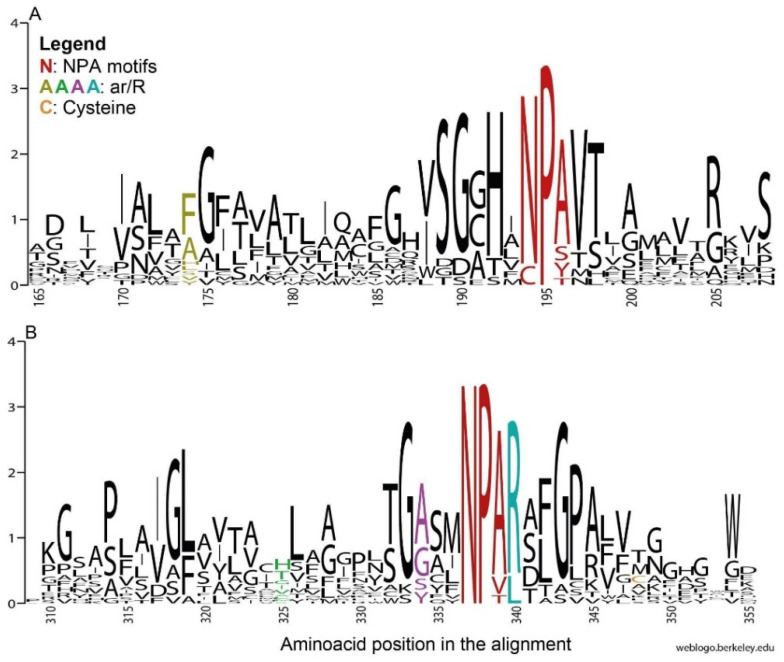
Sequence logos displaying conservation of residues created for all aligned sequences of AQPs (Supplementary Table S3), representing the regions of the first (**A**) and second (**B**) NPA motifs. The y axis represents the probability of the residue occurring at that position. The webserver WebLogo [61] was used.

**Figure 3:**
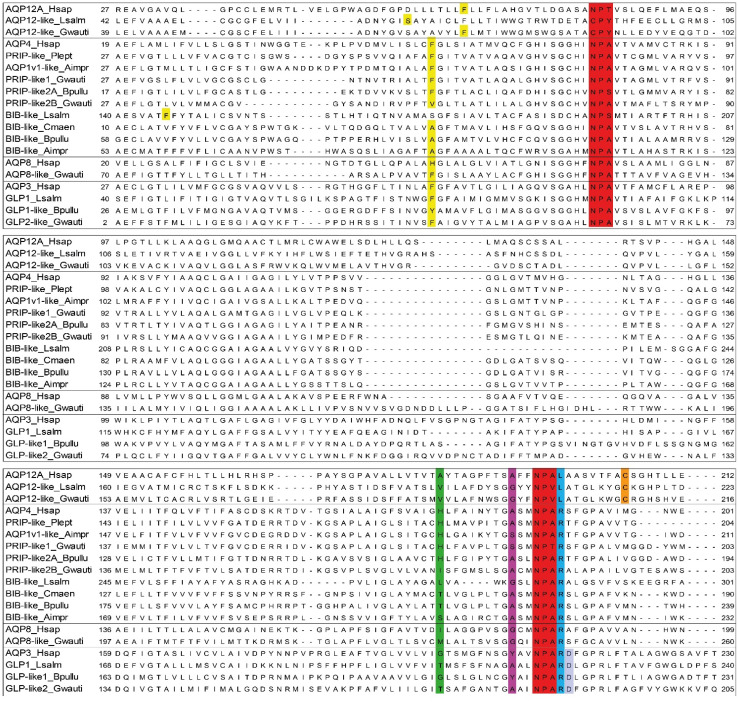
Molecular secondary structures of aquaporins. Multiple alignment of NPA regions of the amphipod (Gwauti: *Gammarus wautieri*; Bpullu: *Baikalogammarus pullus*) proteins with human (Hsap), copepod (Lsalm), balanid (Aimpr), and decapods (Cmaen: *Carcinus maenas*; Plept: *Pontastacus leptodactylus*) sequences. In red, the two NPA motifs. The ar/R residues in the different positions have respectively different colors (yellow, green, purple, and light blue respectively). In shaded blue the Asp residue after the second NPA box of the Glp-like. In orange, the Cys residue down in the C-terminus NPA boxes of the Aqp12-like.

**Figure 4:**
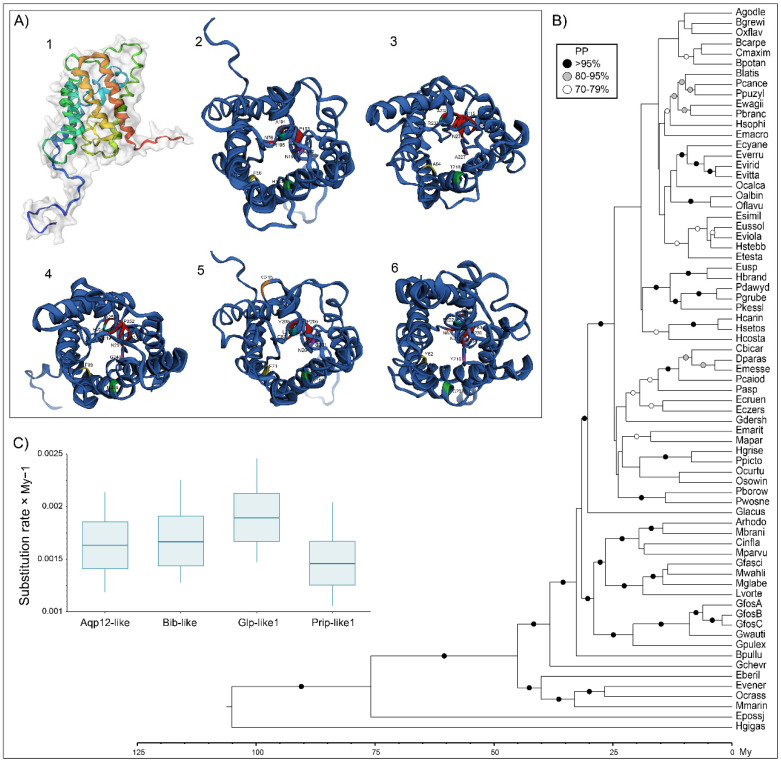
AQPs of gammaroid amphipods. (**A**) Monomers lateral (**A1**) and extracellular (**A2**) cartoon rendering of Prip-like1, Aqp8-like (**A4**), and Aqp12-like (**A5**) of *Gammarus wautieri* (SRR8089730) and extracellular one of Bib-like (**A3**) and Glp-like1 (**A6**) of *Baikalogammarus pullus* (SRR3467045). The ar/R and NPA motifs have the same colors as in Figure 1. (**B**) Calibrated Bayesian phylogeny of nucleotide alignments of gammaroidean Prip-like1 (65), Bib-like (38), Aqp12-like (33), Glp-like1 (62), and COX1 (63). The phylogeny was rooted with *Hirondellea gigas* (Birstein and Vinogradov, 1955). Only nodes with posterior probability >70% display support with circles (black >95, gray 80–95, white 70–79). Complete names, accession numbers, and alignment of the sequences used can be accessed in the Supplementary material (Table S2 and Alignments S2–S6). (**C**) Mean substitution rates relative to COXI (substitution per site × My − 1) for 4 studied genes.

The overall primary (Figure 2 and Figure 3) and tertiary (Figure 4A) structures of gammaroid AQPs appeared similar to those of mammalian AQPs. As experimental studies on the tertiary structure of invertebrate AQPs, especially for Aqp8 and Aqp12, are lacking, these simulations need to be interpreted with caution. However, their structures presented the typical hourglass shape of AQPs, with six domains, two NPA motifs positioned at the end of the two hemihelices, and five loops A-E (Figure 4A; [60]).

#### 3.2.1. NPA Motifs

In general, the NPA box appeared conserved in the gammaroids (Figure 2), and only a few deviations were recovered: Prip-like 1 (*Gammarus wautieri*), Prip-like 2A (*Baikalogammarus pullus*), and Aqp12-like (*G. wautieri*; Figure 3). The third position in the box is the less conserved, and both the NPA deviations in the *N*- and the C-terminals displayed by the Prip-like sequences (i.e., Ser and Thr instead of Ala in the first and the second NPA, respectively) are already known in the literature (e.g., [15,62]). The substitution of Ala with Ser is widespread among both plants and animals (e.g., [62]), and according to our *in silico* analysis, it should not affect the size of the pore. In contrast, the presence of Thr instead of Ala in the C-terminal may affect the structure, reducing the size of the pore and being able to form hydrogen bonds [63]. This mutation is more frequent in the *N*-terminal motif, occurring mostly among the superaquaporins of animals, and in the SIPs (i.e., small and basic intrinsic proteins), an exclusive clade of the plants [62,64]. The gammaroid superaquaporins displayed a divergent CPY motif in the *N*-terminal and Cys in the ninth position after the second NPA box. These substitutions (Asn with Cys and Ala with Tyr) seem to be exclusive to arthropods, having been recovered only in insects (e.g., *Drosophila melanogaster* Meigen 1830, *Chilo suppressalis* (Walker, 1863)), the salmon louse *Leopeophtheirus salmonis,* and the barnacle *Amphibalanus improvisus* (e.g., [15,16,65]. *In silico* analysis pointed out that the exchange of Asn for Cys in the NPA region may have structural and functional consequences because of their hydrophilic and hydrophobic properties, respectively. Additionally, superaquaporins found in *A. improvisus*, from other *in silico* analyses, seem to have reduced permeability to solutes because of the presence of Arg and Tyr residues close to the constriction site [15]. However, the function of these AQPs in arthropods is still not clear.

#### 3.2.2. Ar/R Region

The ar/R residues were variable in general, but there were only some specific substitutions when the single AQP group was taken into account. The fourth position was the most conserved among the ar/Rs, except for Aqp12-like, which displayed Leu instead of Arg. It is supposed that Arg is directly involved in proton exclusion, together with NPA in C-terminal; however, its substitution may or may not affect the water permeability of the protein [66,67]. The ar/R region of the gammaroid Aqp12-like proteins highly diverged from that of humans, but in general, it was conserved through the gammaroids and the other arthropods analyzed in this work (Figure 3, Supplementary S1).

All putative Prips (i.e., Prip-like 1-2A-2B) showed a similar ar/R region to human Aqp4. Only Prip-like 2B from *G. wautieri* presented a substitution in the first two positions, with Val and Ile instead of Phe and His (Figure 2 and Figure 3). The exchange of His with Ile in the second position was also recovered in the Glps of *A. improvisus* and hypothesized that it might lead to an increase in pore size [15]. In fact, His residue is usually present in AQPs permeable only to water, and it is often substituted in mammalian Glps with Gly or Ala. This replacement, together with one of the Cys residues in the third position of ar/R with Tyr or Phe, makes the protein permeable to glycerol and urea and reduces its water permeability [7,66].

The ar/R of Glp-like 1 was similar to that of human Aqp3, while Glp-like 2 displayed the same residues as *L. salmonis* (Figure 3). Similar to the second position of the ar/R of human Glps, the gammaroids showed small residues, i.e., Gly and Thr (in Glp-like 1 and Glp-like 2, respectively). Moreover, the third position presented Tyr (Glp-like 1) and Ala (Glp-like 2), in common with humans and *L. salmonis*, respectively. Finally, similar to most of the Aqp3-like genes, both gammaroid Glp-like genes presented an Asp following the last residue of the selectivity filter. The differences recovered in these two paralogs may suggest a different permeability to water and glycerol, as well as to urea. In fact, the substitutions Gly and Tyr may render the channel more polar, allowing moderately rapid transit of both glycerol and water [7].

As recovered in the ML phylogeny, the gammaroid Bib-like sequences were more similar to the sequences of *A. improvisus* and the crab *Carcinus maenas* than to the Bib of *L. salmonis* (Figure 3). The replacement of Thr with Ser in the ar/R second position of the barnacle does not seem to affect the structure of the protein. Bib proteins, unlike most of the AQPs, seem to be completely intracellular [68]. Being involved in neurogenic activities, such as an increase in the number of sensory organ precursors [69], could make this gene a valid model for adaptations to diverse and extreme environments (e.g., caves and deep-sea).

To our knowledge, this is the first time that putative orthologs of human Aqp8 were found in arthropods. Nonetheless, urea transit was recovered in a few Prips and Glps, showing the importance of this function (e.g., [15,16,70]). However, differently to the Aqp1-like and the Glps, gammaroid Aqp8 presented neither the Hist in the second position of the ar/R nor the Asp after the second NPA underlining its different nature. Comparing humans and gammaroids, ar/R residues differed in the first position, where His was replaced by Phe, and in the second, where Ile was replaced by Met or Leu (Figure 3 and Supplementary Alignment S1). According to [71], substitutions of the first ar/R residue seem to not affect solute exclusion, replacements of Ile in the second position had amino acids with similar properties (i.e., hydrophobic, aliphatic), and *in silico* analysis did not recover any damage to the structure [63]. Moreover, the Gly in the third position, which seems to generate a urea permeable channel [71], was conserved in the gammaroid putative AQP8-like protein. However, it is important to remember that the solute permeability is not determined only by the single ar/R residues but is the result of their interplay with the physical size and the chemical properties of the filter created by these amino acids [71]. Further research is necessary to corroborate the in silico analyses and identify the physiological function of these putative gammaroid genes.

### 3.3. Use of AQPs for Phylogenetic Inferences: A Case Study of Gammaroid Amphipods

Reconstruction of ML phylogenies based on nucleotide sequences of each gene showed general congruence among AQPs for well-supported clades (Supplementary Figures S2–S6). The calibrated Bayesian tree of the four gammaroid AQPs plus COX1 (Figure 4) was generally in accordance with the most recent phylogenies published on this superfamily [46,72]. Unfortunately, the relationships among most of the families of the non-Gammaridae amphipods from Baikal Lake (i.e., above Glacus in Figure 4B) were unclear. This suggests that evolutionary processes of AQP genes require more studies in this group and can show interesting patterns related to radiation in deep ancient lakes. Nevertheless, the phylogeny was well resolved, showing high support (>95%), especially in deeper parts. Additionally, the general temporal framework (both means and 95% HPD ranges, see Supplementary Figure S7) matched other studies on Gammaroidea [46,73]. The obtained rates of substitutions for AQPs, ranging from ca. 0.0015/My-1 (Prip-like1) to ca. 0.0019/My-1 (Glp-like1), are in line with rates obtained for Amphipoda nuclear genes, e.g., 28S rRNA gene (i.e., from 0.0016 [73] to 0.003 [28]), but faster than the 18S rRNA gene (i.e., 0.00068 [73]). These findings suggest that the AQPs can be helpful for phylogenetic reconstructions and molecular clock calibration. However, our results may be biased by the lack of sequences for some genes (Aqp12-like 33 sequences and Prip-like1 65) as well as the lack of overlap in a few sequences in COX1, which was also visible in the ML of the gene (i.e., extremely long branch of *Macropereiopus parvus* Bazikalova, 1945; Supplementary Figure S6). However, with the provision of more data (i.e., species and gene fragments) and once these candidate genes are properly characterized, the AQPs seem to have potential in solving taxonomic issues, especially at deeper taxonomic levels.

## 4. Conclusions

AQPs are fundamental for the proper functioning of cells, and a complete catalog of roles played by these proteins is far from complete. With our *in silico* approach, we found, in the available data of amphipods, potential candidate proteins for all the AQPs already known for crustaceans, including the first record of a putative Aqp8-like. The sequences displayed similar structures to the better characterized AQPs of humans, copepods, and barnacles, with some specific substitutions in the NPA motifs and ar/R residues, in each AQP subfamily, that might modify their substrate selectivity. Furthermore, our phylogenetic analyses showed that the candidate genes coding for these proteins are evolving at a similar pace as conservative nuclear genes, and although they might not be effective in analyses involving recent divergences, they can be useful in solving deeper nodes of phylogenies in the future. The presence of a diverse set of AQPs in animals inhabiting completely different environments, such as amphipods, spanning from coral reefs to urban ponds and encompassing extreme environments such as deep sea or dunal zones, after a proper description and characterization of their AQPs, might turn these organisms into a great model for better understanding the evolutionary processes behind speciation and adaptation to various conditions.

## Data Availability

The data used in this study are openly available in the Supplementary Materials.

## Supplementary Materials

The following are available online at https://www.mdpi.com/article/10.3390/cells10123417/s1, Figure S1: Maximum likelihood phylogeny of translated nonredundant sequences of AQPs, Figure S2: RAxML phylogeny of gammaroid Prip-Like 1, Figure S3: RAxML phylogeny of gammaroid Bib-like, Figure S4: RAxML phylogeny of gammaroid Aqp12-like, Figure S5: RAxML phylogeny of gammaroid Glp-like 1, Figure S6: RAxML phylogeny of gammaroid COX1, Figure S7: Calibrated bayesian tree fully annotated, Table S1: Information on the sequences used for the ML phylogenetic analyses, Table S2: Accession numbers of the nucleotide sequences used for the phylogenetic reconstruction of the Gammaroidea superfamily. Alignment S1: AQP translated sequence alignment, Alignment S2: Nucleotide Aqp12-like of Gammaroida, Alignment S3: Nucleotide Bib-like of Gammaroida, Alignment S4: Nucleotide Prip-like1 of Gammaroida, Alignment S5: Nucleotide Glp-like1 of Gammaroida, Alignment S6: Nucleotide COX1 of Gammaroida, Alignment S7: AQP Alignment for conserved regions and cartoon rendering.

## References

[1] Laloux T., Junqueira B., Maistriaux L.C., Ahmed J., Jurkiewicz A., Chaumont F. (2018). Plant and Mammal Aquaporins: Same but Different. Int. J. Mol. Sci..

[2] Michenkova M., Taki S., Blosser M.C., Hwang H.J., Kowatz T., Moss F.J., Occhipinti R., Qin X., Sen S., Shinn E. (2021). Carbon dioxide transport across membranes. Interface Focus.

[3] Shapiguzov A.Y. (2004). Aquaporins: Structure, Systematics, and Regulatory Features. Russ. J. Plant Physiol..

[4] Fu D., Libson A., Larry J.W.M., Weitzman C., Nollert P., Krucinski J., Robert M.S. (2000). Structure of a Glycerol-Conducting Channel and the Basis for Its Selectivity. Science.

[5] Hub J.S., de Groot B.L. (2008). Mechanism of selectivity in aquaporins and aquaglyceroporins. Proc. Natl. Acad. Sci. USA.

[6] Murata K., Mitsuoka K., Hirai T., Walz T., Agre P., Heymann J.B., Engel A., Fujiyoshi Y. (2000). Structural determinants of water permeation through aquaporin-1. Nature.

[7] Sui H., Han B.-G., Lee J.K., Walian P., Jap B.K. (2001). Structural basis of water-specific transport through the AQP1 water channel. Nature.

[8] Ishibashi K. (2006). Aquaporin subfamily with unusual NPA boxes. Biochim. Biophys. Acta (BBA)-Biomembr..

[9] Abascal F., Irisarri I., Zardoya R. (2014). Diversity and evolution of membrane intrinsic proteins. Biochim. Biophys. Acta (BBA)-Gen. Subj..

[10] Soto G., Alleva K., Amodeo G., Muschietti J., Ayub N.D. (2012). New insight into the evolution of aquaporins from flowering plants and vertebrates: Orthologous identification and functional transfer is possible. Gene.

[11] Ishibashi K., Tanaka Y., Morishita Y. (2020). Chapter One—Perspectives on the evolution of aquaporin superfamily. Vitam. Horm..

[12] Benoit J.B., Hansen I.A., Szuter E.M., Drake L.L., Burnett D.L., Attardo G.M. (2014). Emerging roles of aquaporins in relation to the physiology of blood-feeding arthropods. J. Comp. Physiol. B.

[13] Drake L.L., Boudko D.Y., Marinotti O., Carpenter V.K., Dawe A.L., Hansen I.A. (2011). The Aquaporin Gene Family of the Yellow Fever Mosquito, *Aedes aegypti*. PLoS ONE.

[14] Peng J., Zhou Y., Jia H., Li L., Qian J., Han F., Yin H., Cui Y. (2018). Transcriptomics-Based Identification of Aquaporin Diversity in the House Dust Mite *Dermatophagoides farinae* (Acariformes: Pyroglyphidae). J. Insect Sci..

[15] Lind U., Järvå M., Rosenblad M.A., Pingitore P., Karlsson E., Wrange A.-L., Kamdal E., Sundell K., André C., Jonsson P.R. (2017). Analysis of aquaporins from the euryhaline barnacle *Balanus improvisus* reveals differential expression in response to changes in salinity. PLoS ONE.

[16] Stavang J.A., Chauvigné F., Kongshaug H., Cerdà J., Nilsen F., Finn R.N. (2015). Phylogenomic and functional analyses of salmon lice aquaporins uncover the molecular diversity of the superfamily in Arthropoda. BMC Genom..

[17] Finn R.N., Cerdà J. (2015). Evolution and Functional Diversity of Aquaporins. Biol. Bull..

[18] Kaufmann N., Mathai J.C., Hill W.G., Dow J.A.T., Zeidel M.L., Brodsky J.L. (2005). Developmental expression and biophysical characterization of a *Drosophila melanogaster* aquaporin. Am. J. Physiol.-Cell Physiol..

[19] Kikawada T., Saito A., Kanamori Y., Fujita M., Śnigórska K., Watanabe M., Okuda T. (2008). Dehydration-inducible changes in expression of two aquaporins in the sleeping chironomid, *Polypedilum vanderplanki*. Biochim. Biophys. Acta (BBA)-Biomembr..

[20] Yanochko G.M., Yool A.J. (2002). Regulated Cationic Channel Function in *Xenopus* Oocytes Expressing *Drosophila* Big Brain. J. Neurosci..

[21] Finn R.N., Chauvigné F., Stavang J.A., Belles X., Cerdà J. (2015). Insect glycerol transporters evolved by functional co-option and gene replacement. Nat. Commun..

[22] Niu J., Hu X.L., Ip J.C.H., Ma K.Y., Tang Y., Wang Y., Qin J., Qiu J.-W., Chan T.F., Chu K.H. (2020). Multi-omic approach provides insights into osmoregulation and osmoconformation of the crab *Scylla paramamosain*. Sci. Rep..

[23] Rahi M.L., Moshtaghi A., Mather P.B., Hurwood D.A. (2018). Osmoregulation in decapod crustaceans: Physiological and genomic perspectives. Hydrobiologia.

[24] Horton T., Lowry J., De Broyer C., Bellan-Santini D., Coleman C.O., Corbari L., Costello M.J., Daneliya M., Dauvin J.-C., Fišer C. (2021). World Amphipoda Database. http://www.marinespecies.org/amphipoda.

[25] Väinölä R., Witt J.D.S., Grabowski M., Bradbury J.H., Jazdzewski K., Sket B. (2008). Global diversity of amphipods (Amphipoda; Crustacea) in freshwater. Hydrobiologia.

[26] MacNeil C., Dick J.T.A., Platvoet D., Briffa M. (2011). Direct and indirect effects of species displacements: An invading freshwater amphipod can disrupt leaf-litter processing and shredder efficiency. J. North Am. Benthol. Soc..

[27] MacNeil C., Dick J.T.A., Elwood R.W. (1999). The dynamics of predation on *Gammarus* spp. (Crustacea: Amphipoda). Biol. Rev..

[28] Mamos T., Wattier R., Burzyński A., Grabowski M. (2016). The legacy of a vanished sea: A high level of diversification within a European freshwater amphipod species complex driven by 15 My of Paratethys regression. Mol. Ecol..

[29] Wildish D.J. (1982). Evolutionary ecology of reproduction in gammaridean Amphipoda. Int. J. Invertebr. Reprod..

[30] Grabowski M., Mamos T., Bącela-Spychalska K., Rewicz T., Wattier R.A. (2017). Neogene paleogeography provides context for understanding the origin and spatial distribution of cryptic diversity in a widespread Balkan freshwater amphipod. PeerJ.

[31] Wattier R., Mamos T., Copilaş-Ciocianu D., Jelić M., Ollivier A., Chaumot A., Danger M., Felten V., Piscart C., Žganec K. (2020). Continental-scale patterns of hyper-cryptic diversity within the freshwater model taxon *Gammarus fossarum* (Crustacea, Amphipoda). Sci. Rep..

[32] Desiderato A., Costa F.O., Serejo C.S., Abbiati M., Queiroga H., Vieira P.E. (2019). Macaronesian islands as promoters of diversification in amphipods: The remarkable case of the family *Hyalidae* (Crustacea, Amphipoda). Zool. Scr..

[33] Grabowski M., Wysocka A., Mamos T. (2017). Molecular species delimitation methods provide new insight into taxonomy of the endemic gammarid species flock from the ancient Lake Ohrid. Zool. J. Linn. Soc..

[34] Gesteira J.L.G., Dauvin J.C. (2000). Amphipods are Good Bioindicators of the Impact of Oil Spills on Soft-Bottom Macrobenthic Communities. Mar. Pollut. Bull..

[35] Kunz P.Y., Kienle C., Gerhardt A., Whitacre D.M. (2010). *Gammarus* spp. in Aquatic Ecotoxicology and Water Quality Assessment: Toward Integrated Multilevel Tests. Reviews of Environmental Contamination and Toxicology Volume 205.

[36] Moore P.G., Rainbow P.S., Hayes E. (1991). The beach-hopper *Orchestia gammarellus* (Crustacea: Amphipoda) as a biomonitor for copper and zinc: North Sea trials. Sci. Total. Environ..

[37] Feckler A., Thielsch A., Schwenk K., Schulz R., Bundschuh M. (2012). Differences in the sensitivity among cryptic lineages of the *Gammarus fossarum* complex. Sci. Total. Environ..

[38] Soucek D.J., Dickinson A., Major K.M., McEwen A.R. (2013). Effect of test duration and feeding on relative sensitivity of genetically distinct clades of *Hyalella azteca*. Ecotoxicology.

[39] Conlan K.E., Desiderato A., Beermann J. (2021). *Jassa* (Crustacea: Amphipoda): A new morphological and molecular assessment of the genus. Zootaxa.

[40] Marchini A., Cardeccia A. (2017). Alien amphipods in a sea of troubles: Cryptogenic species, unresolved taxonomy and overlooked introductions. Mar. Biol..

[41] Cabezas M.P., Xavier R., Branco M., Santos A.M., Guerra-García J.M. (2014). Invasion history of *Caprella scaura* Templeton, 1836 (Amphipoda: Caprellidae) in the Iberian Peninsula: Multiple introductions revealed by mitochondrial sequence data. Biol. Invasions.

[42] Rewicz T., Wattier R., Grabowski M., Rigaud T., Bacela-Spychalska K. (2015). Out of the Black Sea: Phylogeography of the invasive killer shrimp *Dikerogammarus villosus* across Europe. PLoS ONE.

[43] Catalán-García M., Chauvigné F., Stavang J.A., Nilsen F., Cerdà J., Finn R.N. (2021). Lineage-level divergence of copepod glycerol transporters and the emergence of isoform-specific trafficking regulation. Commun. Biol..

[44] Katoh K., Standley D.M. (2013). MAFFT Multiple Sequence Alignment Software Version 7: Improvements in Performance and Usability. Mol. Biol. Evol..

[45] Geneious 11.1. https://www.geneious.com.

[46] Mamos T., Grabowski M., Rewicz T., Bojko J., Strapagiel D., Burzyński A. (2021). Mitochondrial Genomes, Phylogenetic Associations, and SNP Recovery for the Key Invasive Ponto-Caspian Amphipods in Europe. Int. J. Mol. Sci..

[47] Pomianowski K., Burzyński A., Kulczykowska E. (2021). A de novo Transcriptome Assembly of the European Flounder (Platichthys flesus): The Preselection of Transcripts Encoding Active Forms of Enzymes. Front. Mar. Sci..

[48] Guindon S., Dufayard J.-F., Lefort V., Anisimova M., Hordijk W., Gascuel O. (2010). New Algorithms and Methods to Estimate Maximum-Likelihood Phylogenies: Assessing the Performance of PhyML 3.0. Syst. Biol..

[49] Anisimova M., Gascuel O. (2006). Approximate Likelihood-Ratio Test for Branches: A Fast, Accurate, and Powerful Alternative. Syst. Biol..

[50] Lefort V., Longueville J.-E., Gascuel O. (2017). SMS: Smart Model Selection in PhyML. Mol. Biol. Evol..

[51] Stamatakis A. (2014). RAxML version 8: A tool for phylogenetic analysis and post-analysis of large phylogenies. Bioinformatics.

[52] Bouckaert R., Vaughan T.G., Barido-Sottani J., Duchêne S., Fourment M., Gavryushkina A., Heled J., Jones G., Kühnert D., De Maio N. (2019). BEAST 2.5: An advanced software platform for Bayesian evolutionary analysis. PLOS Comput. Biol..

[53] Copilaş-Ciocianu D., Sidorov D., Gontcharov A. (2019). Adrift across tectonic plates: Molecular phylogenetics supports the ancient Laurasian origin of old limnic crangonyctid amphipods. Org. Divers. Evol..

[54] Copilaş-Ciocianu D., Zimţa A.-A., Grabowski M., Petrusek A. (2018). Survival in northern microrefugia in an endemic Carpathian gammarid (Crustacea: Amphipoda). Zool. Scr..

[55] Mamos T., Jażdżewski K., Čiamporová-Zaťovičová Z., Čiampor F.J., Grabowski M. (2021). Fuzzy species borders of glacial survivalists in the Carpathian biodiversity hotspot revealed using a multimarker approach. Sci. Rep..

[56] Rambaut A., Drummond A.J., Xie D., Baele G., Suchard M.A. (2018). Posterior Summarization in Bayesian Phylogenetics Using Tracer 1.7. Syst. Biol..

[57] Kelley L.A., Mezulis S., Yates C.M., Wass M.N., Sternberg M.J.E. (2015). The Phyre2 web portal for protein modeling, prediction and analysis. Nat. Protoc..

[58] Reynolds C.R., Islam S.A., Sternberg M.J.E. (2018). EzMol: A Web Server Wizard for the Rapid Visualization and Image Production of Protein and Nucleic Acid Structures. J. Mol. Biol..

[59] Ittisoponpisan S., Islam S.A., Khanna T., Alhuzimi E., David A., Sternberg M.J.E. (2019). Can Predicted Protein 3D Structures Provide Reliable Insights into whether Missense Variants Are Disease Associated?. J. Mol. Biol..

[60] Verkman A.S., Yang B., Song Y., Manley G.T., Ma T. (2000). Role of water channels in fluid transport studied by phenotype analysis of aquaporin knockout mice. Exp. Physiol..

[61] WebLogo. https://weblogo.berkeley.edu/.

[62] Ishibashi K., Morishita Y., Tanaka Y., Yang B. (2017). The Evolutionary Aspects of Aquaporin Family. Aquaporins.

[63] Betts M.J., Russell R.B. (2003). Amino Acid Properties and Consequences of Substitutions. Bioinform. Genet..

[64] Danielson J.Å., Johanson U. (2008). Unexpected complexity of the Aquaporin gene family in the moss *Physcomitrella patens*. BMC Plant Biol..

[65] Lu M.-X., Pan D.-D., Xu J., Liu Y., Wang G.-R., Du Y.-Z. (2018). Identification and Functional Analysis of the First Aquaporin from Striped Stem Borer, *Chilo suppressalis*. Front. Physiol..

[66] Beitz E., Wu B., Holm L.M., Schultz J.E., Zeuthen T. (2006). Point mutations in the aromatic/arginine region in aquaporin 1 allow passage of urea, glycerol, ammonia, and protons. Proc. Natl. Acad. Sci. USA.

[67] Kitchen P., Conner A.C. (2015). Control of the Aquaporin-4 Channel Water Permeability by Structural Dynamics of Aromatic/Arginine Selectivity Filter Residues. Biochemistry.

[68] Rao Y., Jan L.Y., Jan Y.N. (1990). Similarity of the product of the *Drosophila* neurogenic gene big brain to transmembrane channel proteins. Nature.

[69] Doherty D., Jan L.Y., Jan Y.N. (1997). The *Drosophila* neurogenic gene big brain, which encodes a membrane-associated protein, acts cell autonomously and can act synergistically with Notch and Delta. Development.

[70] Herraiz A., Chauvigné F., Cerdà J., Bellés X., Piulachs M.-D. (2011). Identification and functional characterization of an ovarian aquaporin from the cockroach *Blattella germanica* L. (Dictyoptera, Blattellidae). J. Exp. Biol..

[71] Kitchen P., Salman M.M., Pickel S.U., Jennings J., Törnroth-Horsefield S., Conner M.T., Bill R.M., Conner A.C. (2019). Water channel pore size determines exclusion properties but not solute selectivity. Sci. Rep..

[72] Naumenko S.A., Logacheva M.D., Popova N.V., Klepikova A.V., Penin A.A., Bazykin G.A., Etingova A.E., Mugue N.S., Kondrashov A.S., Yampolsky L.Y. (2017). Transcriptome-based phylogeny of endemic Lake Baikal amphipod species flock: Fast speciation accompanied by frequent episodes of positive selection. Mol. Ecol..

[73] Copilaş-Ciocianu D., Borko Š., Fišer C. (2020). The late blooming amphipods: Global change promoted post-Jurassic ecological radiation despite Palaeozoic origin. Mol. Phylogenetics Evol..

