# Supplementary figures and images for: First Glimpse at the Diverse Aquaporins of Amphipod Crustaceans
Source: Cells. 2021 Dec 4;10(12):3417. doi: 10.3390/cells10123417 (PMC8699810; doi:10.3390/cells10123417)

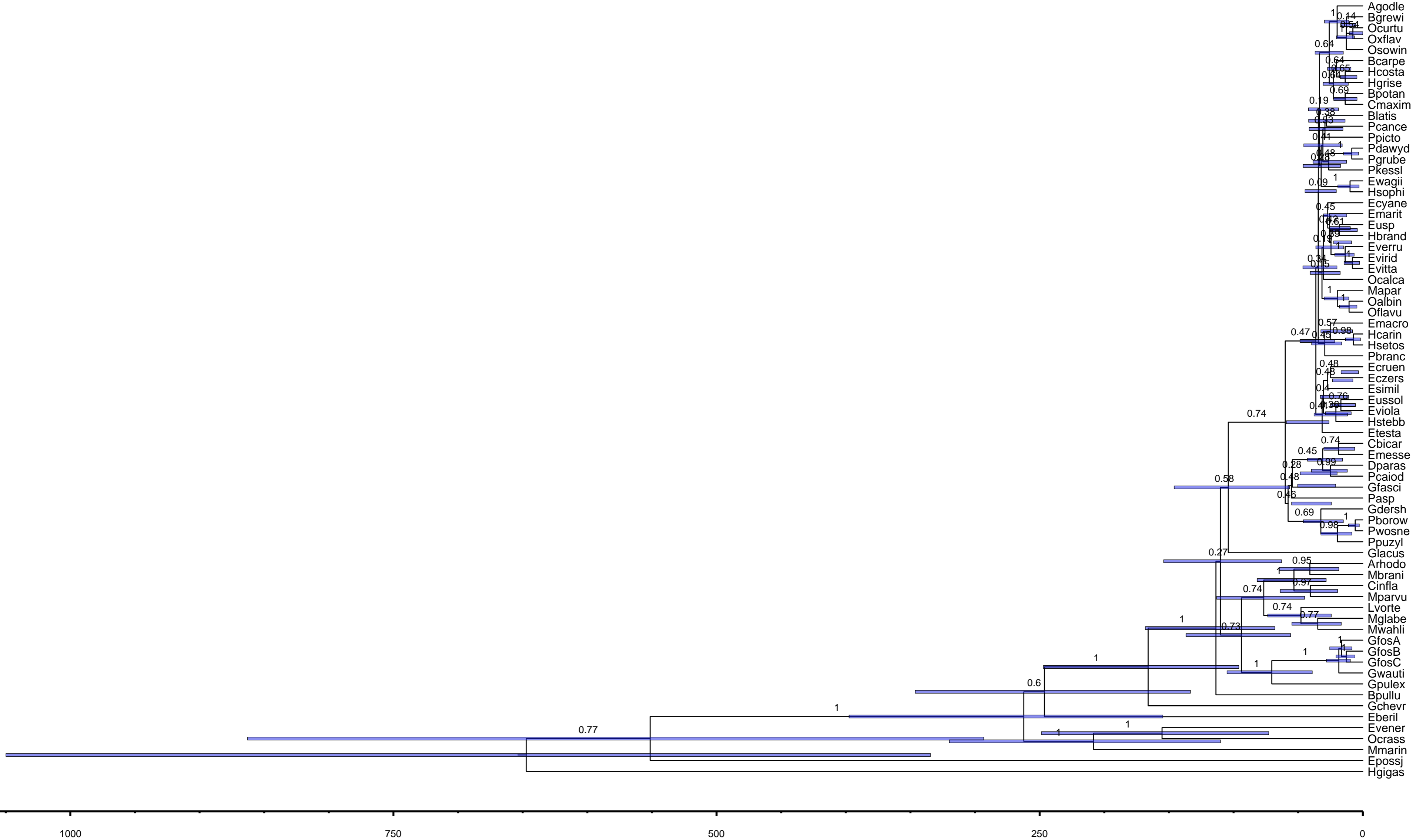

Supplement: Supplementary file 1 [file cells-10-03417-s001.zip › Figures_suppl/Figure S2 Calibrated bayesian tree fully annotated. Only posterior probabilities higher than 70% are shown. Bars on nodes referring to 95% HPD.pdf]

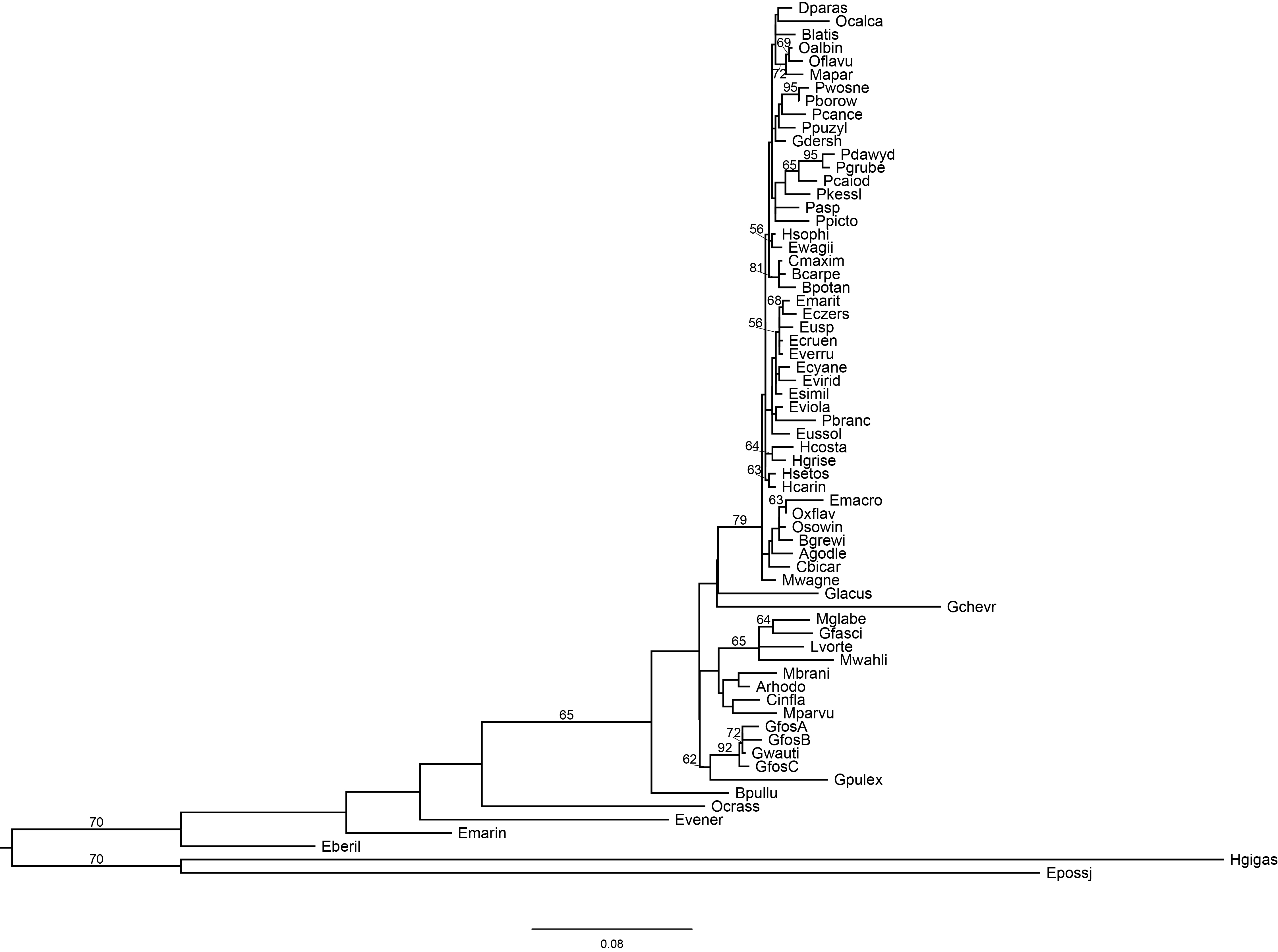

Supplement: Supplementary file 1 [file cells-10-03417-s001.zip › Figures_suppl/Figure S2. RAxML phylogeny of gammaroid Prip-Like 1.jpg]

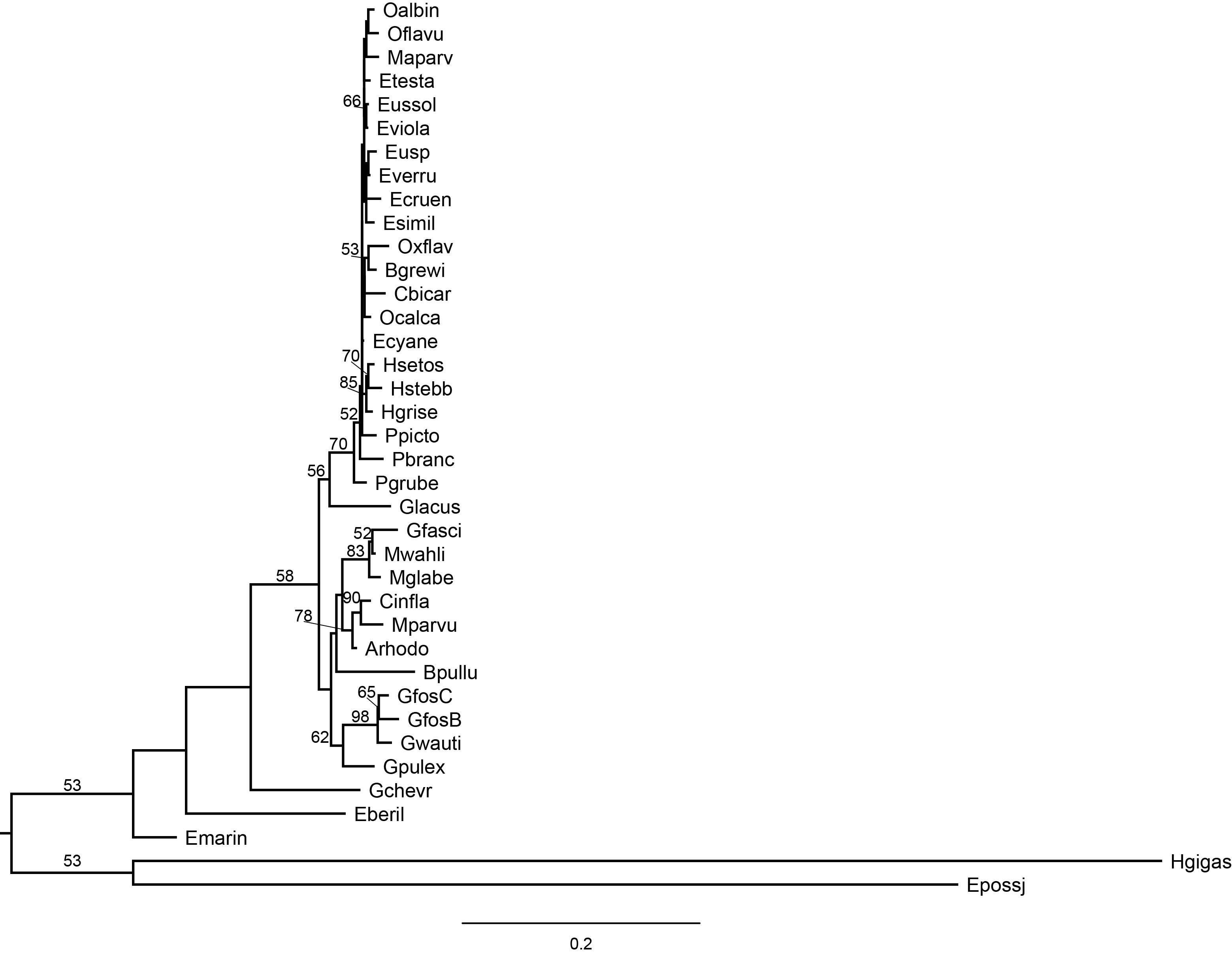

Supplement: Supplementary file 1 [file cells-10-03417-s001.zip › Figures_suppl/Figure S3 RAxML phylogeny of gammaroid Bib-like.jpg]

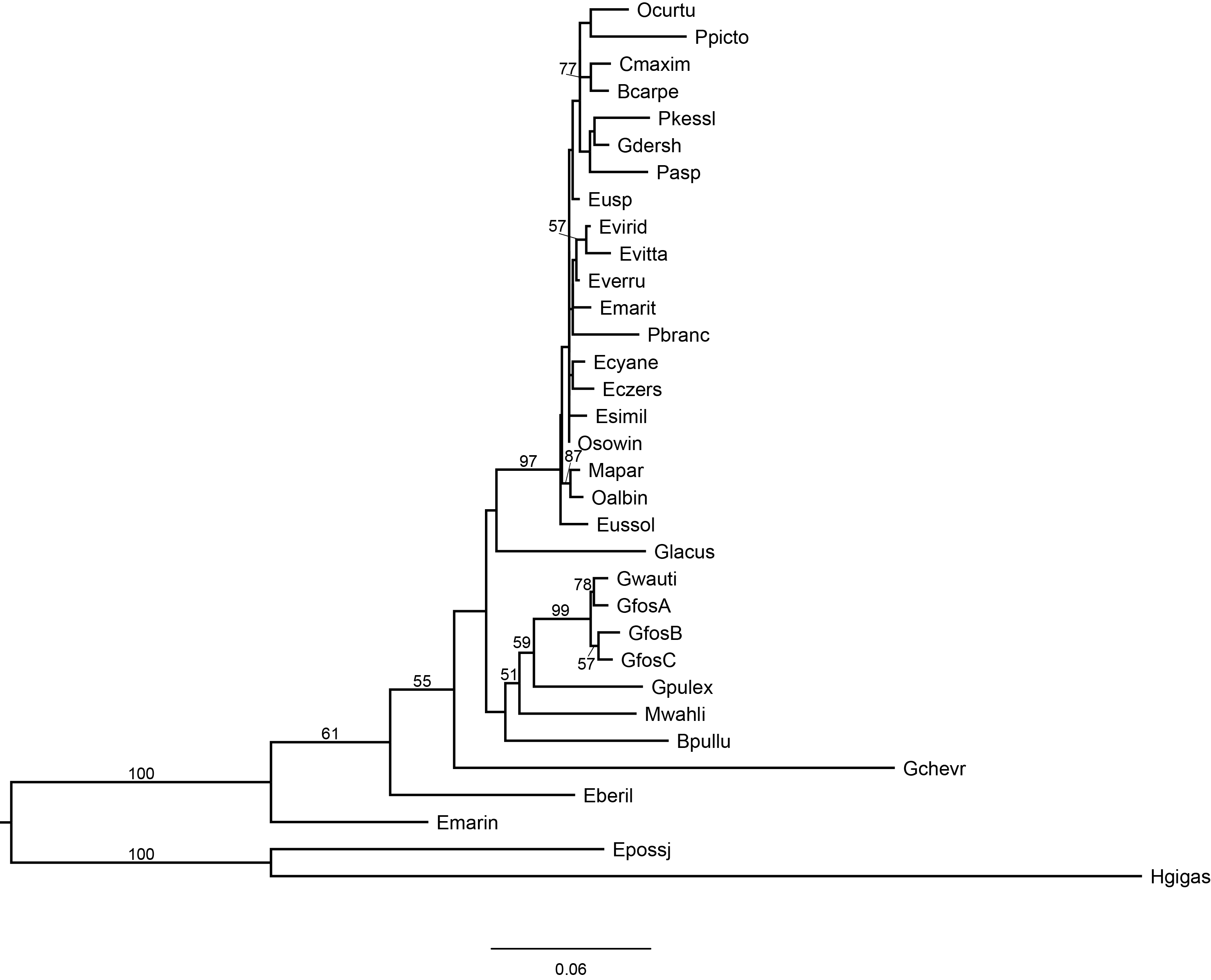

Supplement: Supplementary file 1 [file cells-10-03417-s001.zip › Figures_suppl/Figure S4 RAxML phylogeny of gammaroid Aqp12-like.jpg]

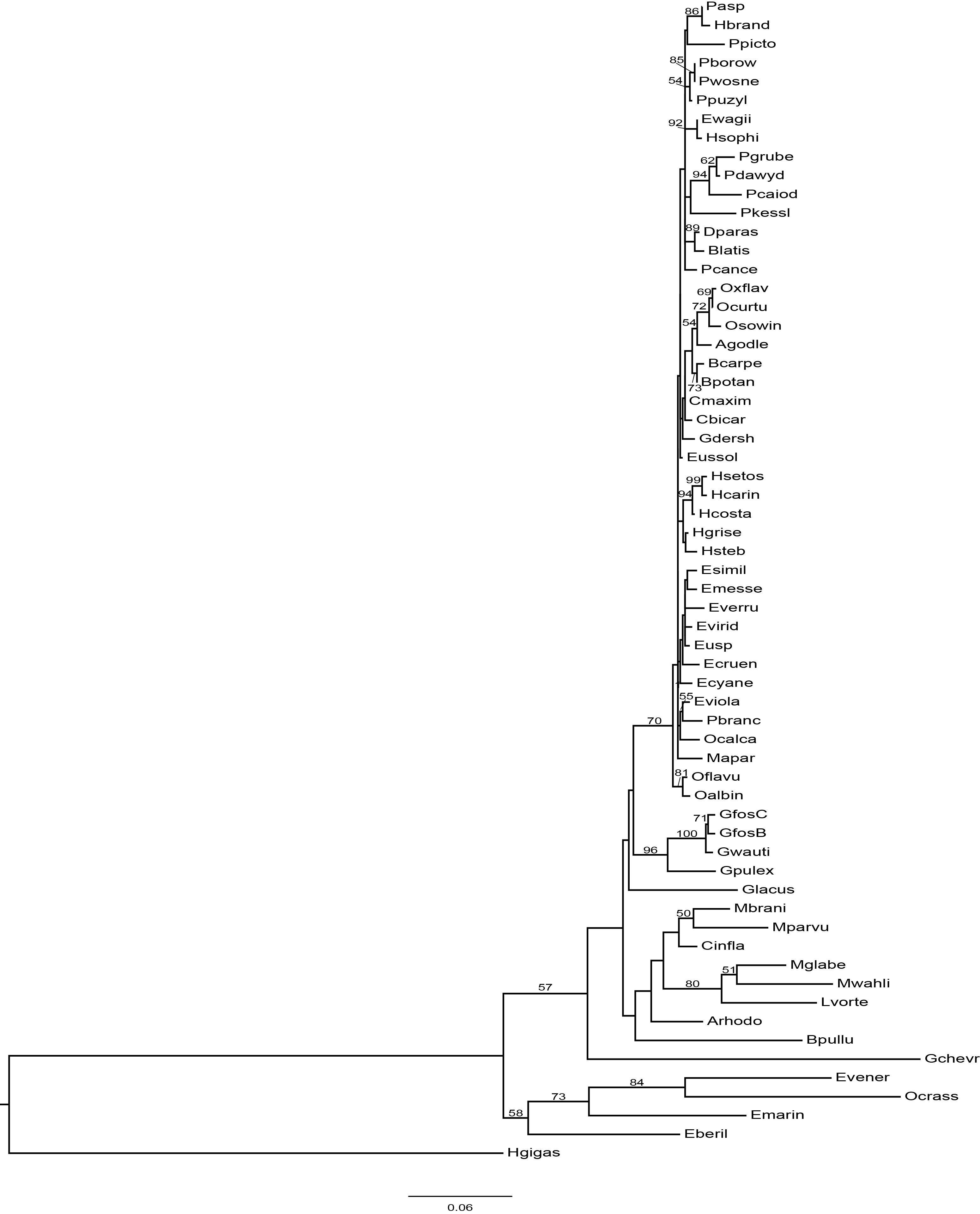

Supplement: Supplementary file 1 [file cells-10-03417-s001.zip › Figures_suppl/Figure S6. RAxML phylogeny of gammaroid COX1.jpg]

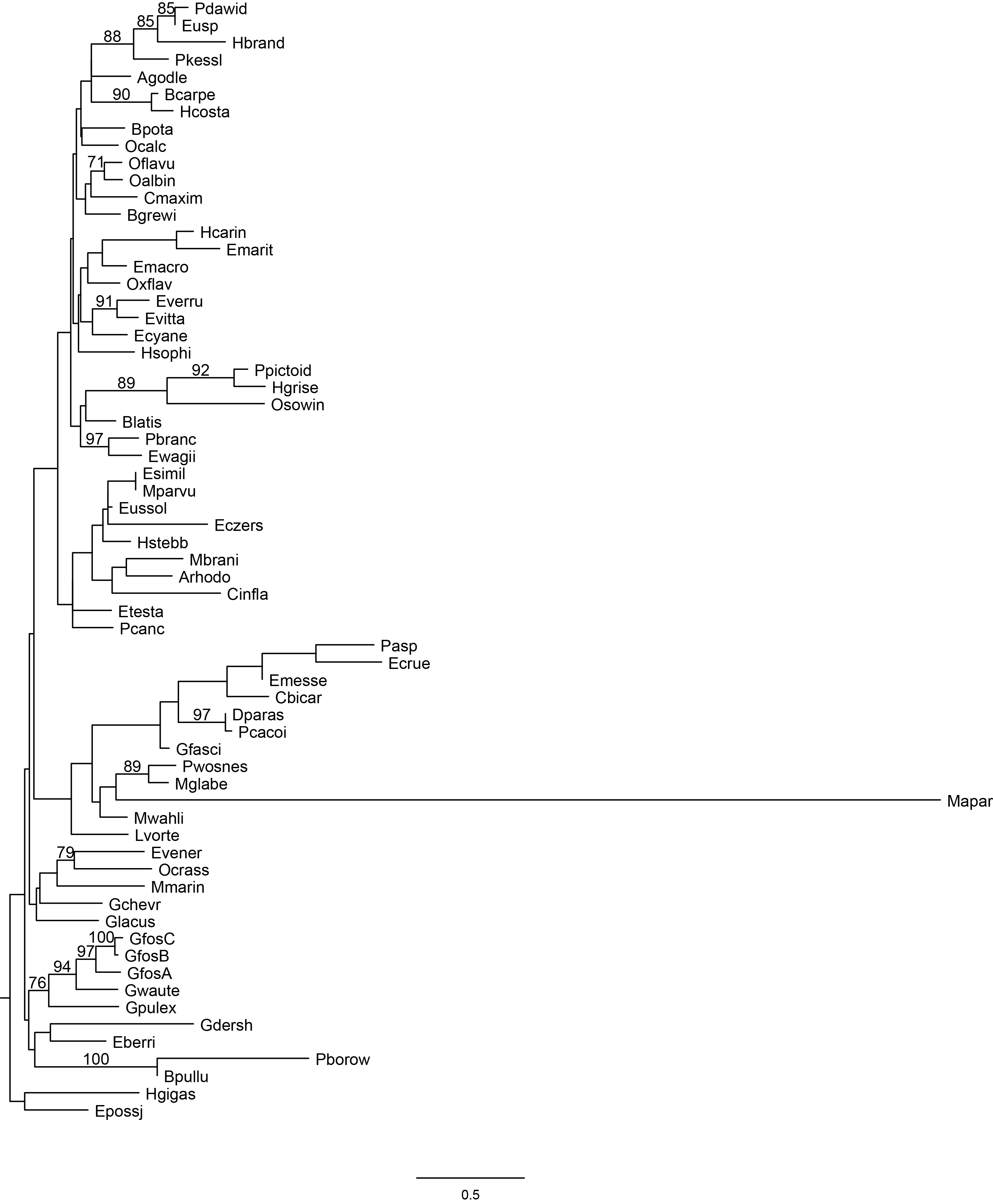

Supplement: Supplementary file 1 [file cells-10-03417-s001.zip › Figures_suppl/RAxML_bipartitions.COX1_Alignment_N_1.tre.jpg]
