# Supplementary material for: First Glimpse at the Diverse Aquaporins of Amphipod Crustaceans
Source: Cells. 2021 Dec 4;10(12):3417. doi: 10.3390/cells10123417 (PMC8699810; doi:10.3390/cells10123417)

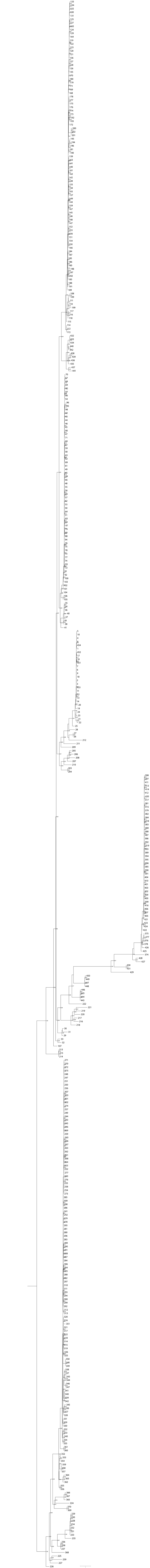

Supplement: Supplementary file 1 [file Figure S1 Maximum likelihood phylogeny of translated non-redundant sequences of AQPs.pdf › Figures_suppl/Figure S1 Maximum likelihood phylogeny of translated non-redundant sequences of AQPs.pdf]
